# Modeling the potential impacts of climate change and adaptation strategies on groundnut production in India

**DOI:** 10.1016/j.scitotenv.2021.145996

**Published:** 2021-07-01

**Authors:** M.D.M. Kadiyala, Swamikannu Nedumaran, Jyosthnaa Padmanabhan, Murali Krishna Gumma, Sridhar Gummadi, Srinivas Reddy Srigiri, Richard Robertson, Anthony Whitbread

**Affiliations:** a Acharya NG Ranga Agricultural University, Guntur, India; b International Crops Research Institute for the Semi-Arid Tropics (ICRISAT), Patancheru 502 324, Andhra Pradesh, India; c International Food Policy Research Institute, 2033 K St NW, Washington, DC 20006, USA; d German Development Institute, Bonn, Germany; e International Rice Research Institute, Viet Nam

**Keywords:** Groundnut, DSSAT, Climate change, Adaptation, Spatial modeling

## Abstract

Groundnut is one of the significant sources of oil, food, and fodder in India. It is grown in marginal arid and semi-arid agro-ecosystems with wide yield fluctuations due to spatial variability of rainfall and soil. Climate change, which is predicted to increase the intra- and inter-annual rainfall variability will further constrain the groundnut economy in India besides the global and domestic economic, social and policy changes. Through this study we aim to examine the biophysical and social economic impacts of climate change on groundnut production and prices to provide a comprehensive analysis of how agriculture and the food system will be affected. Using projected climate data for India, we estimated the biophysical impacts of climate change on groundnut during mid-century using representative concentration pathway (RCP 8.5) scenario. We examined the impacts of changes in population and income besides environmental factors on groundnut productivity. This is to highlight the importance of holistic assessment of biophysical and socioeconomic factors to better understand climate change impacts. Modelled projections show that by 2050, climate change under an optimistic scenario will result in −2.3 to 43.2% change in groundnut yields across various regions in India when climate alone was factored in. But the change in groundnut yields ranged from −0.9% to 16.2% when economic (population and income) and market variables (elasticities, trade, etc.) were also considered. Similarly, under pessimistic climate change scenario, the percent change in groundnut yields would be −33.7 to 3.4 with only the climate factored in and −11.2 to 4.3 with the additional economic and market variables included. This indicates the sensitivity of climate change impacts to differences in socioeconomic factors. This study highlights the need to take into account market effects to gain a holistic understanding of how economic and environmental factors impact agricultural food systems and economies.

## Highlights

• Projected climate change will impact groundnut yields by −34 to 43% across India.
• Adaptation synergies developed showed promising in adverse climate conditions.
• Biophysical and economic model integration is crucial for assessment of climate change.

## Graphical abstract

**Figure:**
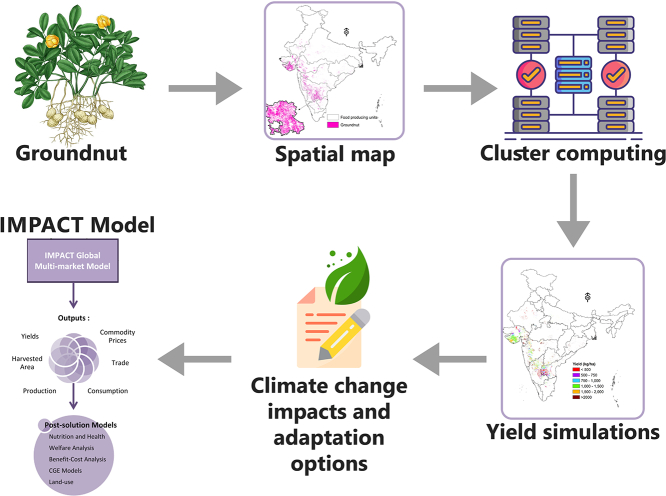

## 1 Introduction

Agriculture and allied sectors are important sources of livelihood for most of the 833 million people residing in rural India (Census of India, 2011). Agriculture contributes 17.3% to the Gross Domestic Product (GDP). Although this is less compared to the service sector's contribution at 53.6% (Sector wise Contribution of GDP of India, 2019), agriculture plays a critical role in food, nutritional security and rural employment. India remains committed to maintaining food security for its population through various government programs.

Indian agriculture faces a serious threat from climate change that may lead to severe challenges in ensuring food security for its growing population. Climate change impacts are expected to reduce food production, leading to higher food prices, reduced consumption and increased number of people with malnourishment and hunger (Nelson et al., 2014; Pingali et al., 2019). Several researchers have studied the impacts of climate change on various crops and their productivity using different methodological approaches. They have used either process-based biophysical models or economic and statistical models (Singh et al., 2014a; Rosenzweig and Hillel, 2015; Kadiyala et al., 2015; Cammarano et al., 2020). Crop simulation and other autoregressive models have been widely used to examine the impacts of abiotic stresses such as long-term changes in surface temperatures, carbon dioxide (CO_2_) emissions and rainfall on various crops (Halder et al., 2020; Ye et al., 2020; Demirhan, 2020).

Groundnut (*Arachis hypogaea L*.) is an important food and oilseed crop grown in India across varying agro-climatic environments. It is mostly grown (83% of total groundnut area) under rainfed conditions during the monsoon season (June/July to October/November) and the remaining 17% is grown under irrigated conditions in the post-monsoon (October–March) season (Singh et al., 2014b). Globally and in India many biotic and abiotic stresses limit the groundnut productivity. However, heat and drought stress have been observed to be the main factors limiting the yield (Prasad et al., 2009a, Prasad et al., 2009b). Temperatures during the crop growing period were already close to or above the upper limit of the optimum temperature range (20–30 °C) required for the crop (Weiss, 2000). The projected temperature changes for these regions in the coming years will intensify heat and drought stresses in groundnut, further limiting productivity.

Recent studies using crop simulation models to examine groundnut yields in 2050 report as much as a 25% decrease in yields compared to 2010 (Singh et al., 2014b). However, these results were obtained without considering technological changes and impacts of long-run drivers such as growth in incomes, area, production and crop yields. Over the years, new crop technologies may have been adopted by farmers in response to changes in population, income, in addition to climate. These factors should be considered while assessing impacts of climate change on various crops and cropping systems as they have a significant effect on consumption and expenditure on food commodities. Globally, by 2050 yields of major crops such as maize, rice, wheat, and soybean will be around 11% lower due to the effects of climate change combined with economic responses. When compared with climate alone, the yield decline will be 25% (Islam et al., 2016). These studies indicate the importance of linking climate, crop and economic models to understand the magnitude and trade-offs of climate change.

However, there is very little evidence so far to show how climate change and variability over the next few decades will impact groundnut productivity and production in a particular country and its impact on price and net trade globally. It is also essential to understand if one major groundnut producing country plans to adopt adaptation measures and if the measures have an impact on groundnut prices locally and globally. While researchers have quantified the impacts of climate change on several crops and regions, most of the studies have focused on assessing biophysical impacts without considering economic factors and future pathways. Therefore, it is critical to understand how climate change impacts will influence developing countries adaptation and mitigation measures and assess the impacts on crop production and prices.

This study

a) assessed the potential biophysical and socioeconomic impacts of climate change on groundnut production spatially across India; and.
b) studied the impacts on groundnut production, price and net trade with different adaptation options to mitigate climate change impacts.

## 2 Materials and methods

This study used a structural modeling approach developed by International Food Policy Research Institute (IFPRI) under the Global Futures and Strategic Foresight program. In this approach, we integrated a geospatially coordinated suite of biophysical, socioeconomic models with process-based biophysical and partial equilibrium economic models (Islam et al., 2016; Robinson et al., 2015a, Robinson et al., 2015b) to assess the impact of climate change on groundnut in India.

### 2.1 Biophysical model – DSSAT crop model

The CROPGRO-groundnut model was used to evaluate climate change impacts and adaptation options such as agronomic and improved genetic traits of groundnut for increasing its productivity under both current and future climates in the target sites. The CROPGRO-groundnut model is part of a suite of crop models available in decision support system for agrotechnology transfer (DSSAT) version 4.5 software (Hoogenboom et al., 2010) was used extensively by the researchers. In this model, high temperatures affect the crop growth and development with the allocation of assimilates to the reproductive organs resulting in decreased pod set and seed growth (Prasad et al., 2003). The CROPGRO model's ability to not only accurately predict high temperature effects (Kumar et al., 2012; Singh et al., 2012; Halder et al., 2020) but also leaf and canopy assimilation responses to CO_2_ seen in soybean (Alagarswamy et al., 2006) and groundnut (Bannayan et al., 2009; Halder et al., 2015; Rajwade et al., 2015) makes it an ideal choice to study climate change impacts in groundnut. In the model, we used future climate data during the mid-century period (2040-69), and the corresponding projected CO_2_ concentration (571 ppm) according to RCP 8.5 to study the climate change impacts.

### 2.2 Spatial modeling - groundnut crop type mapping

The terra moderate resolution imaging spectroradiometer (MODIS) vegetation indices (MOD13Q1) version 5 data are generated every 16 days. Imagery was acquired from the Land Processes Distributed Active Archive Center (LP DAAC) (https://lpdaac.usgs.gov/). Image classification was performed by rescaling 16-day normalized difference vegetation index (NDVI) images and later stacking them into a single data composite for each cropping year (Gumma et al., 2016; Thenkabail et al., 2007). In this study, monthly NDVI maximum value composites (MVCs) were used for cataloging. An NDVI 16-day composite was used for identifying and labelling land use/land cover classes, including groundnut areas in India. Class identification and labelling were done based on decision tree algorithms, and spectral matching techniques along with intensive ground survey data.

### 2.3 Climate data and models

Climate data for each simulation grid cell over India (5 arc minutes) were obtained from the Intergovernmental Panel on Climate Change's 5th assessment datasets (IPCC, 2014). This database provided all the required climatic elements needed by the stochastic daily weather generator SIMMETEO available in DSSAT. The weather generator provides daily weather consistent with the monthly averages supplied to it from the FutureClim data (Hijmans et al., 2005; Jones and Thornton, 2003, Jones and Thornton, 2013). The baseline climate we used for this study was that of 1980 to 2009 as suggested in agricultural model intercomparison and improvement program (AgMIP) regional integrated assessment protocols (Rosenzweig and Hillel, 2015). We then took the future climates from the AgMIP/The Inter-Sectoral Impact Model Intercomparison Project (ISIMIP) downscaling with differences extracted and perturbed to the trusted FutureClim data. Projected climate data for 2050 (2040–2069) for RCP 8.5 taken from Geophysical Fluid Science laboratory's Earth System Modeling (GFDL-ESM2M) (Dunne et al., 2012a; Dunne et al., 2012b) and Hadley Centre's Global Environment Model (HadGEM 2-ES) version 2 (Martin et al., 2011). We selected these GCM's because they appeared, on an average, across the globe, to be the driest and wettest. They therefore provide two extreme climates to compare the impacts of climate change on groundnut in India.

Among the two GCMs, HadGEM2-ES is the driest (Wiebe et al., 2015) and was therefore selected because groundnut is mostly grown under rainfed conditions and is sensitive to water stress. In this paper we focused on the RCP 8.5 scenario. Both the GCMs result in higher temperature and rainfall anomalies compared to RCP 6.0 and 4.5 with lower global greenhouse gas emissions (Warszawski et al., 2014). Similarly, we used GFDL-ESM2M to investigate the wettest scenario for climate change in the groundnut. RCP 8.5 scenario is treated as a possible scenario if society does not make concerted efforts to cut greenhouse gas emissions. The reason behind selecting two forceful scenarios like RCP8.5 is, that it represents the pessimistic scenario and with adaptations towards this outcome, the agricultural sector will be well prepared to adjust to conditions likely under less forcing scenarios.

### 2.4 Soil profile input data

**Supplementary Fig. 1:**
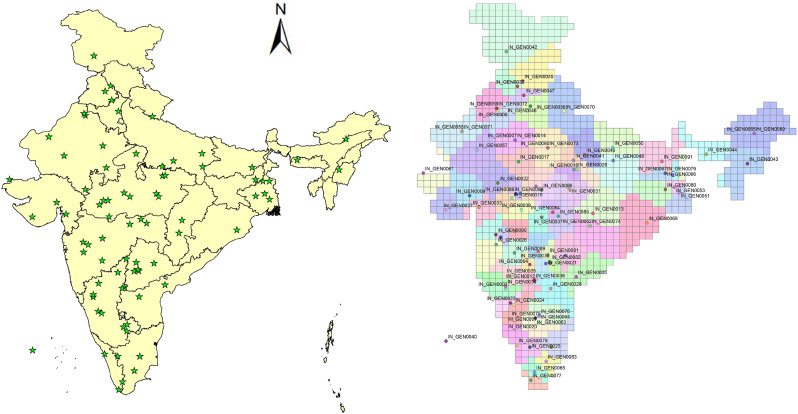
Spatial locations of soil profile data collected from groundnut growing regions of India (left pane) and spatial soil profile map generated (right pane)

Biophysical crop simulation models usually require profile-wise soil data. For each grid cell, we obtained soil inputs from a set of 90 soil profiles developed by blending and interpreting information from crop modeling studies conducted in India in various locations and from the World Inventory of Soil Emission Potentials (WISE) database (Batjes, 2009). The soils mostly consist of Alfisols, Inceptisols, Entisols and Vertisols with detailed soil profile data and soil distribution map provided in the supplementary material (supplementary Fig. 1). We ran crop model simulations for all soils in each grid cell and computed cell-specific output from the area-weighted average based on the area share of each soil in the grid cell. Instead of using available broad generic soil database, we made an effort to collect soil profile data from International Crops Research Institute for the Semi-Arid Tropics (ICRISAT) data repository which were checked for quality and were specific to groundnut growing regions. We derived soil physical and chemical properties such as texture, hydraulic parameters, bulk density, organic matter and available N for each location based on the available soil profile data. Additional soil parameters such as soil albedo, drainage constant, and runoff curve number were estimated based on soil texture and converted using the generic soil database available in the DSSAT-models (Tsuji et al., 1998).

### 2.5 Planting and crop management information

In India, almost all regions typically plant in July for the rainy (June to October monsoon) season. We tested additional simulations by planting a month earlier or later than July which had the highest yield among the three plantings. The cultivar we used in this study was JL24, which we calibrated and evaluated by Singh et al. (2012) based on long term datasets available with ICRISAT during the 1986–1991 seasons and multi-site Initial Variety Trials–II data obtained from annual reports of the All India Coordinated Research Project on Groundnut. We initialized the simulations on 1st January with 200 kg/ha surface residue. Cultivar JL24 was sown at a plant population of 33 plants m^−2^. Nitrogen was applied as a basal dose as per the recommendation (20 kg/ha) at the time of sowing. The simulations were run at 5 arc min resolution scale using High-Performance Computing (HPC) clusters.

### 2.6 Development of adaptation option

We developed virtual cultivars with desirable yield improving plant traits by changing crop life cycle and yield potential traits (Singh et al., 2014a, Singh et al., 2014b). Genetic coefficients determining the maximum leaf photosynthesis rate (LFMAX), maximum fraction of daily growth partitioned to pod (XFRT) and seed-filling duration for pod cohort (SFDUR) of cultivars were increased by 10%. We tried agronomic management options (critical irrigation at 60 DAS) as an adaptation strategy to combat climate change impacts on groundnut production in India. The reason for increasing the capacity of both source (LFMAX) and sink (XFRT and SFDUR) in groundnut was that there was sufficient genetic variation in photosynthesis rate, harvest index and partition intensity. We observed that these factors have a positive association with total sink size and pod yield (Nautiyal et al., 2012). Due to non-senescence nature of groundnut we observed higher yields with increased LFMAX during the development of virtual cultivars. We used 60 mm of irrigation at 60 days after sowing which coincided with the pod development stage. The reason for using 60 mm irrigation depth was based on farmer's feedback during stakeholder consultation meeting.

### 2.7 Estimation of yield changes under different scenarios

We estimated the biophysical impact of climate change spatially across India by calculating changes in biophysical parameters between the baseline and the future climate scenarios. We calculated the changes during the mid-century period (2040–2069) relative to the baseline (1980–2009) for the respective years and impacts of adaptation options as shown below

$$\text{Yield change}=\frac{\mathrm{Yi}-\mathrm{Yb}}{\mathrm{Yb}.}$$

where, Y*i* is the value of the parameter under future climate (2040–2069) and also under adaptation options, Y*b* is the value of the parameter under the baseline climate. We obtained groundnut yields under each food producing unit (FPU) by summing up the grid cell values within the cultivated groundnut area obtained from crop type mapping generated using remote sensing of a given FPU.

### 2.8 Economic modeling framework

Our team assessed the long term projections of supply, demand, trade and prices of agricultural commodities in countries across regions using the International Model for Policy Analysis of Agricultural Commodities and Trade (IMPACT) (Robinson et al., 2015a, Robinson et al., 2015b).

The model uses crop area, prices, and productivity growth (intrinsic productivity growth rates (IPR's)) to determine the supply of 62 food commodities. The IPRs, which are crop and country-specific, summarize the potential improvements over years in agricultural productivity that can come from advances in management practices, crop improvement and agricultural extension. We used elasticity estimates from country-level studies to establish the relationship between supply, demand and price of each commodity in the model. In case of missing elasticity estimates, we used approximations based on values from similar neighboring countries in the region.

In the IMPACT modeling framework, the partial equilibrium economic trade model, which simulates national and international markets, is linked with other models like the water (hydrology, water basin management and water stress models), crop system model (DSSAT), climate models (General Circulation Models (GCMs)), value chain and land-use model. The model equates supply and demand across the globe to determine the world price of individual commodity. It integrates information flows among the component modules in a consistent equilibrium framework that supports longer-term scenario analysis (Robinson et al., 2015a, Robinson et al., 2015b). The daily climate data from GCMs are used as inputs in the crop models to simulate the crop yields. The outputs from crop and hydrology models are used as input into IMPACT as yield drivers to project crop production, demand, trade, among others at global, regional and national scales. The climate and technology-induced crop yield shocks were used as inputs in the agriculture sector economic model (Islam et al., 2016). At each country level, the gap between demand and supply is resolved through international trade, leaving countries as either net importers/exporters. The wedge/difference between the country and world price reflects the effect of market margin and trade policies.

The economic scenarios used in the IMPACT model are based on Shared Socioeconomic Pathways (SSPs) (O'Neill et al., 2014). The SSP's reflect different future assumptions on the country's population and income trends. In this study, we used SSP2 middle-of-the-road scenario described by O'Neill et al., 2014. The data and parametrization of SSPs in IMPACT is provided in the model documentation (Robinson et al., 2015a, Robinson et al., 2015b). Briefly, long–run drivers such as population and income growth (as represented in SSP2) are used as inputs in IMPACT's economic model. SSP2 (middle-of-the-road scenario) which follows historical trends has been considered as baseline scenario. In this scenario economic development continues but it is not uniform. Environmental degradation continues but at a slowing pace. There is a general improvement, but it is much slower than that seen in SSP 1. Climate change presents moderate challenges to both adaptation and mitigation. Summary of GDP, population, pre-determined growth rates of socioeconomic drivers and assumptions used in IMPACT model for SSP2 scenario were presented in Supplementary Tables 2, 3, 4.

These models together help in understanding the market effects of crop production and its response to climate change. This is accomplished by incorporating climate change effects into the crop yield response to the economic model. Using this suite of models, we assessed the impact of climate change on major groundnut growing regions in India, referred to as FPUs in IMPACT terminology. The IMPACT model takes into account 13 FPUs in India which correspond to the major river basins. Since this study is on groundnut, the study focused on the major groundnut producing basins in India, Ganges, Godavari, Indus, Krishna, Luni, and Tapti.

The economic model links crop yields and changes in output prices endogenously. The model assumes that farmers will respond to changes in prices by varying the use of inputs, such as fertilizer, chemicals, and labor, which will have an impact on the yield. Over the years, if the price of a crop declines, farmers have less incentive to allocate resources to that crop, resulting in a decline in crop yields. The biophysical models involve technology and climate and are therefore independent of prices, and these represent “non market” effects. The economic model captures the markets and prices, which are denoted as “market” effects.

## 3 Results

### 3.1 Groundnut areas and simulation yields

**Fig. 1:**
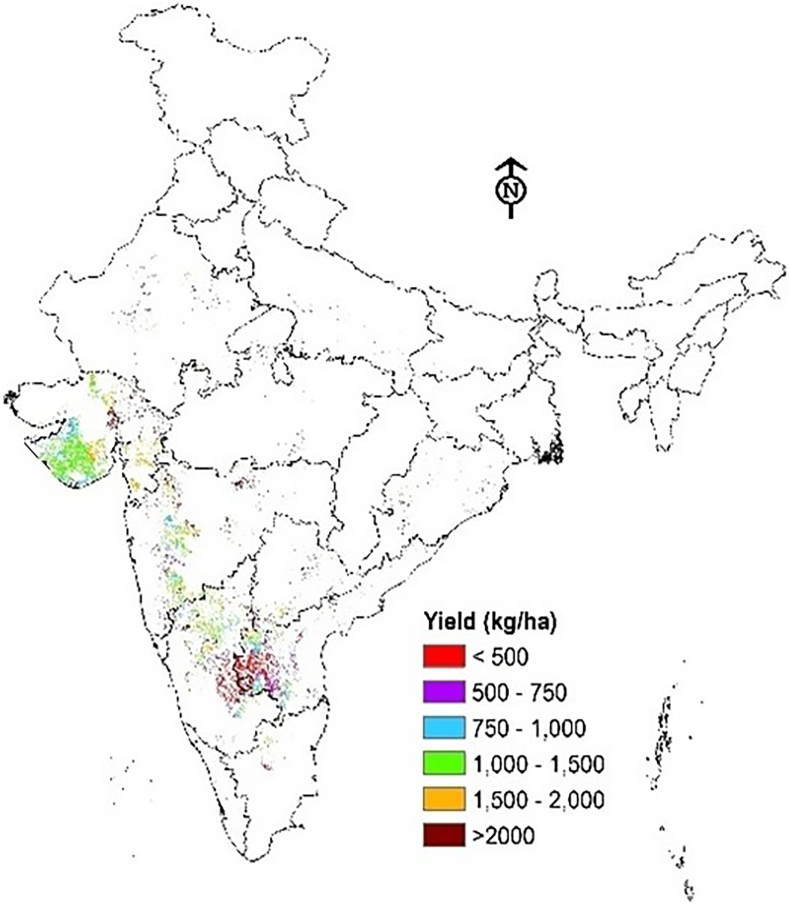
Spatial distribution of groundnut yields across India under base climate.

**Supplementary Fig. 2:**
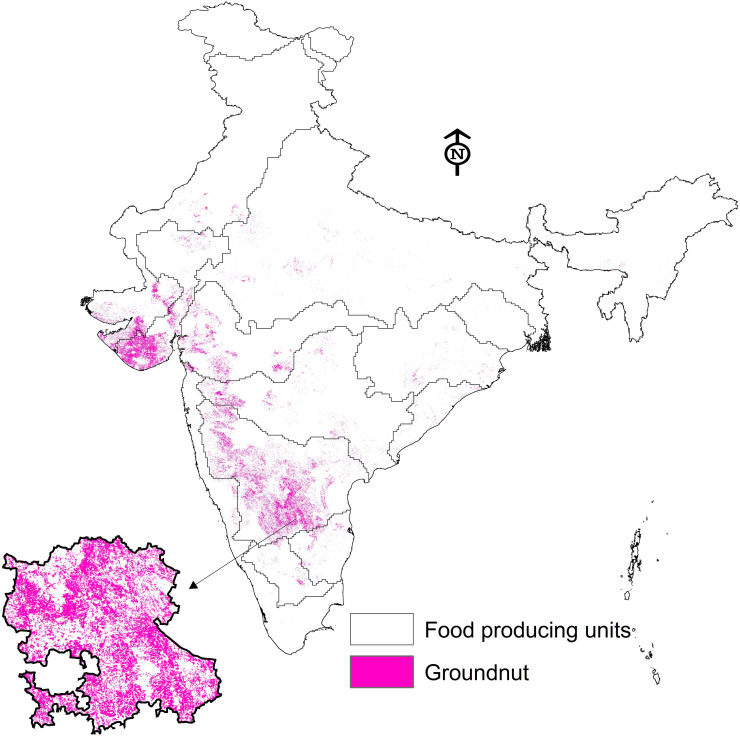
Groundnut crop area in different FPUs in India.

Critical observation of groundnut crop area map developed from satellite imagery (supplementary Fig. 2.) indicates that groundnut in India is mostly grown in the Ganges, Godavari, Indus, Krishna, Luni, and Tapti FPUs. Baseline and future climate yields and impacts of adaptation options were therefore simulated using the DSSAT model for these FPUs only. The base yields (1980–2009) ranged between 794 and 2737 kg/ha, showing spatial heterogeneity across India. The spatial analysis of yield data across the FPUs (Fig. 1) showed that the Ganges FPU had the highest yields due to its high rainfall and soil fertility. The Tapti and Godavari FPUs followed next. The lowest yields were observed in the Indus FPU.

### 3.2 Climate change impacts on groundnut yields

We used two GCMs, one optimistic (HadGEM 2-ES) and another pessimistic (GFDL-ESM2M) to study the impact of climate change on groundnut yields under RCP 8.5 during the mid-century period. The summary of the projected percentage change in groundnut yields between the baseline (1980–2009) and the mid-century future climate (2040–2069) are presented in Table 1. Under climate change, groundnut yields showed a declining trend with the GFDL-ESM2M scenario, and an increasing trend with the HadGEM 2-ES GCMs in a majority of groundnut growing FPUs. Climate change impacts were more prevalent in the Ganges and Indus basins than other FPUs. The yield penalty under pessimistic GCM were as high as −33.7% in the Ganges basin, with overall yields ranging between −33.7% and 12.2% compared to the baseline. Under optimistic GCM, yield improvements were as high as 43.2% in the Godavari FPU and ranged between −2.3 and 43.2%.

**Table 1:** The impact of climate change on rainfed groundnut yields (kg ha^−1^) in different groundnut FPUs of India during mid-century period (2040-2069).

FPU	Base climate (1980–2009)	Climate change (2040–2069)			
		HadGEM 2-ES		GFDL-ESM2M	
	Yield (kg ha^−1^)	Yield (kg ha^−1^)	% change	Yield (kg ha^−1^)	% change
Ganges	2737	2674	−2.3	1814	−33.7
Godavari	1869	2676	43.2	2098	12.2
Indus	1150	1444	25.6	794	−30.9
Krishna	1065	1421	33.4	1098	3.1
Luni	1286	1776	38.1	1329	3.4
Tapti	2109	2451	16.2	2038	−3.4

### 3.3 Response of groundnut yields to adaptation options

#### 3.3.1 Yield boosting cultivar of groundnut

We tested incorporating yield boosting genetic traits by increasing maximum leaf photosynthesis rate, partitioning daily growth to pods, and seed filling duration compared with the baseline cultivar (JL24) as an adaptation option. Incorporating these traits in JL-24 resulted in increased yields under both the GCMs. However, the response was more significant under HadGEM 2-ES in five out of six FPUs. The average yield benefit with yield boosting cultivars under both GCMs across FPUs were 17.1 and 18.1% with GFDL-ESM2M and HadGEM 2-ES respectively (Supplementary Table 1).

#### 3.3.2 Critical irrigation

Groundnut in India is grown under rainfed conditions in most of the groundnut growing areas. However, most of the time, the crop may experience dry spells during pod filling. Providing one critical irrigation under these conditions will be beneficial for crop growth. We tried applying one critical irrigation at 60 DAS as an adaptation option. This resulted in increases in yield under both GCMs and in all the FPUs. However, the response was spatially different across different FPUs. Yield gains from critical irrigation were 24.9% and 34.8% under HadGEM 2-ES and GFDL-ESM2M, respectively in the Krishna basin (supplementary Table 1). The responses were lowest in the Ganges and Luni basins under HadGEM 2-ES. Luni basin was lowest under both HadGEM 2-ES and GFDL-ESM2M GCMs. This underlines the need for location-specific adaptation options.

#### 3.3.3 Combination of yield boost (YB) and critical irrigation (CI)

**Fig. 2:**
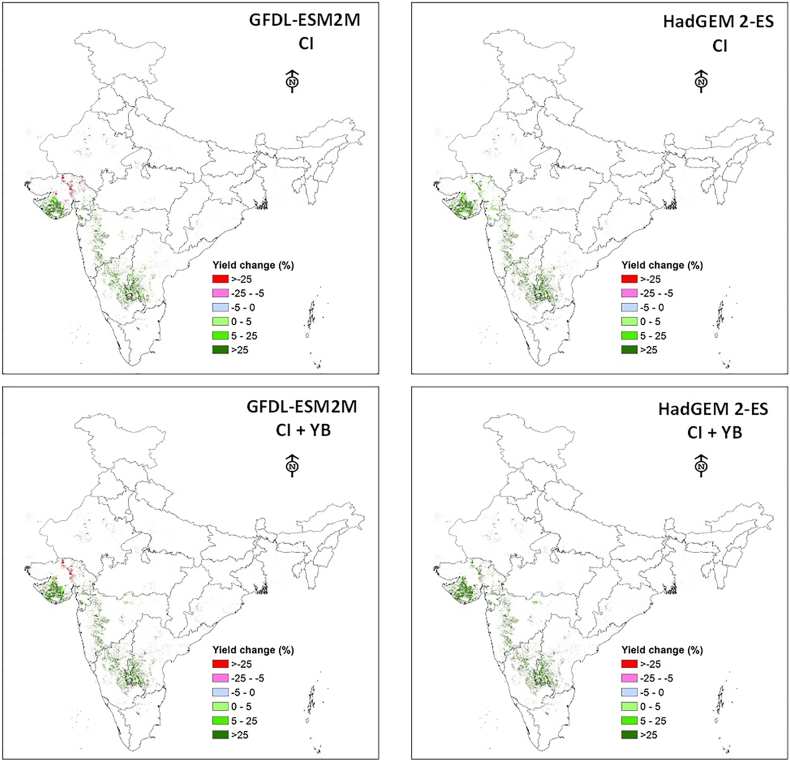
Yield advantages of adaptation options over rainfed groundnut across India under GFDL-ESM2M (left pane) and HadGEM 2-ES (right pane) climate projections.

As the simulation study clearly showed spatially different responses with yield boost and critical irrigation, we tried a simulation of combining both CI and YB using future climate data. The responses were very encouraging across FPUs. The highest response to the combination was observed in the Krishna basin under both the GCMs, with responses ranging between 27% and 54.2% under GFDL-ESM2M and 19.6% and 44.9% with HadGEM 2-ES GCMs (supplementary Table 1). The spatial distribution of yield benefits under various adaptation options across groundnut growing regions are depicted in Fig. 2. Regional aggregation of data indicates that southern India regions experience the highest benefits due to the adoption of both critical irrigation and yield boost cultivars. Further, the response to yield boost cultivar was mostly uniform spatially across locations. However, the response to agronomic management was very prominent in the Krishna basin where the highest area of groundnut was cultivated (Fig. 2). As expected, the responses to adaptation options were more pronounced with optimistic HadGEM 2-ES GCM.

### 3.4 Key results of the economic model on yield, production and net trade of groundnut with market effects in India

#### 3.4.1 Supply, demand, and trade of groundnut in India

**Fig. 3:**
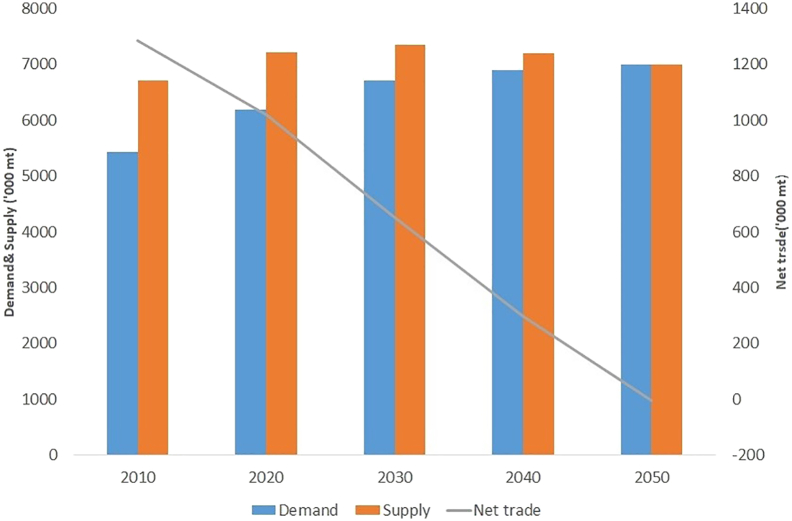
Baseline projections for the supply, demand and net trade in groundnut in India.

Projected supply, demand and net trade of groundnut in India from 2010 to 2050 under a no climate change scenario are presented in Fig. 3. Baseline projections (reference scenario - no climate change) indicate a steady increase in demand for groundnut between 2010 and 2050. Production is also projected to increase between 2010 and 2050 but at a slower rate. The supply-demand gap is estimated to narrow down as production is projected to increase, though not at a pace that would be sufficient to cater to the growth in domestic demand. This is likely to turn India from a net exporter in 2010 to a net importer by 2050, without factoring in climate change impacts.

#### 3.4.2 Impact of climate change on rainfed groundnut yield and production in India

We took both climate change induced effects and market effects on groundnut yields are going to be affected by climate change even the market effects were also taken into consideration. When market effects based on the economic model results are factored in, there is a reduction of more than 50% in the negative impacts on groundnut yields under both climate scenarios in the Ganges, Indus, and Tapti basins (Table 2). This highlights the need to consider market effects to gain a holistic understanding of how both economic and environmental factors impact systems/economies. This is the key in undertaking assessments of a complex phenomenon like climate change to have the right kind of policy making.

**Table 2:** Projected percent rainfed groundnut yield changes under climate change scenarios in mid-century period (2040–2069) across various food producing units.

FPU	HadGEM 2-ES		GFDL-ESM2M	
	Without market effects	With market effects	Without market effects	With market effects
Ganges	−2.3	−0.9	−33.7	−11.2
Godavari	43.2	16.1	12.2	4.3
Indus	25.6	8.9	−30.9	−10.3
Krishna	33.4	12.2	3.1	1.1
Luni	38.1	14.1	3.4	1.2
Tapti	16.2	5.7	−3.4	−1.2

Total annual groundnut production under the baseline scenario is between 23 metric tons (MT) and 354 MT in 2050 across various Indian FPUs Under the HadGEM 2-ES scenario, groundnut production is projected to be higher than the baseline in all FPUs (6.1 to 16.1%), except the Ganges basin (−0.6%) in 2050. Similarly, under the GFDL-ESM2M scenario, production is projected to fall in the Ganges (−11.6%), Indus (−10.5%) and Tapti (−1.3%) FPUs and increase in all other FPUs compared to the baseline in 2050 (Table 3). In both climate scenarios, the majority of the production would come from the Krishna and Luni basins in 2050.

**Table 3:** Groundnut production levels (′000 MT) in major FPUs as influenced by climate change and adaptation strategies in 2050 with market effects.

FPU	Baseline-no climate change	With climate change		Climate change with adaptation					
		GFDL-ESM2M	HadGEM-2-ES	GFDL-ESM2M			HadGEM-2-ES		
				CI	YB	YB+CI	CI	YB	YB+CI
Ganges	32.7	28.9	32.5	27.9	29.0	30.3	32.0	35.8	35.6
Godavari	24.2	25.2	28.1	29.5	28.8	33.5	32.2	35.2	37.2
Indus	150.7	134.9	164.7	132.9	134.1	142.7	179.4	197.0	205.7
Krishna	353.8	357.6	397.7	438.9	391.5	489.2	509.0	477.3	586.9
Luni	246.1	249.0	281.3	263.2	275.1	291.3	306.3	345.0	349.1
Tapti	23.1	22.8	24.5	24.4	25.0	27.2	25.4	28.2	28.5

#### 3.4.3 Adaptation options for increasing yield and production of groundnut in India under different climate scenarios with market effects

##### 3.4.3.1 HadGEM 2-ES scenario

**Fig. 4:**
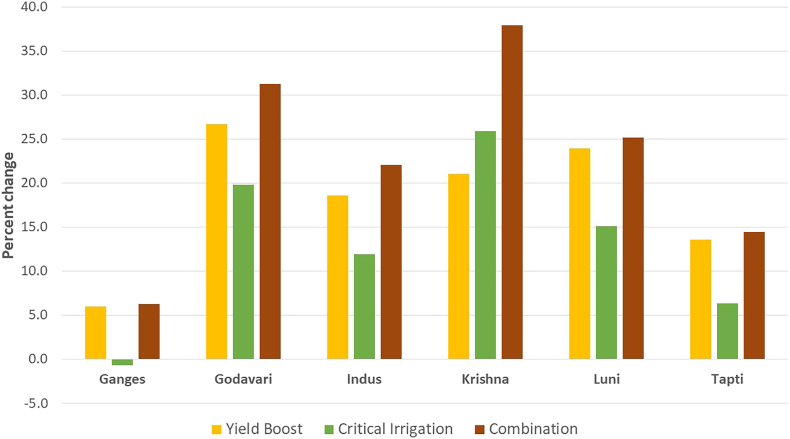
Projected percent rainfed groundnut yields changes under different adaptation strategies in HadGEM 2-ES scenarios in 2050.

Groundnut yields increased under all adaptation options. To the greatest extent, under the adaptation scenario in which, higher-yielding cultivars were clubbed with critical irrigation in all FPUs in 2050 compared to baseline under the HadGEM 2-ES scenario. Yield increases ranged between 6% in the Ganges basin to 38% in the Krishna basin. Adopting high-yielding cultivars alone is projected to increase yields in all the basins but to a lesser extent, ranging between 6% in the Ganges basin and 27% in the Godavari basin compared to baseline. Providing one critical irrigation is also projected to increase yields in all FPUs in 2050, except in the Ganges basin under the HadGEM 2-ES scenario. However, the projected increase is low in both the adaptation options and ranges between 6% to 26% in the Tapti and Krishna basins, respectively (Fig. 4).

Groundnut production under the HadGEM 2-ES scenario is higher under all adaptation options. To the greatest extent under the adaptation scenario in which higher yielding cultivars are used combination with critical irrigation in all FPUs compared to the baseline scenario. The increases in production range between 9% in the Ganges basin to 66% in the Krishna basin in 2050 compared to baseline. Using high yielding cultivars is also projected to increase production in all the basins in 2050 but to a lesser extent, ranging between 9% in the Ganges basin to 46% in the Godavari basin. Similarly, one irrigation during crop growth period projected to result in increased yields in all FPUs except for the Ganges basin in 2050 under the HadGEM 2-ES scenario. The magnitude of increase is projected to be lesser than the other adaptation options and ranges between 10% in the Tapti basin to 44% in the Krishna basin (Table 3).

##### 3.4.3.2 GFDL-ESM2M scenario

**Fig. 5:**
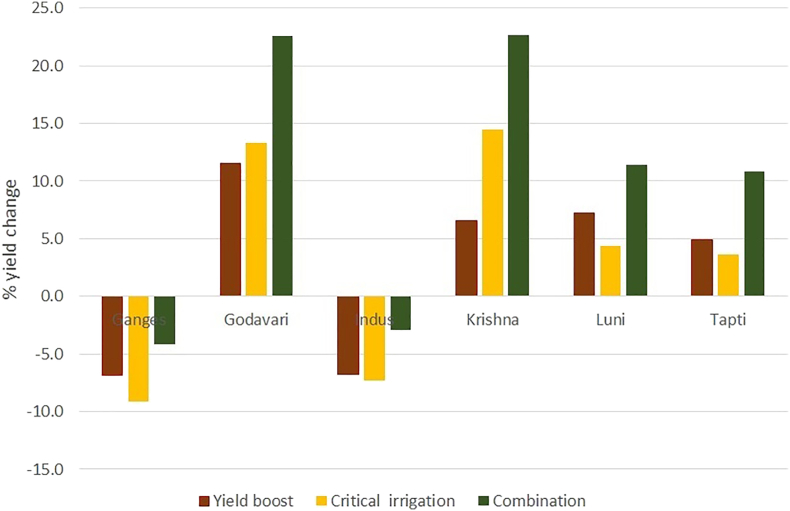
Projected percent change in rainfed groundnut yields using different adaptation strategies under GFDL-ESM2M scenarios in 2050.

Groundnut yields increased under all adaptation options. The highest increase is in the option of combining high yielding cultivars with critical irrigation in all FPUs compared to baseline in 2050, under the GFDL-ESM2M scenario, similar to the HadGEM 2-ES scenario. The increase ranged between 11% in the Tapti basin to 23% in the Krishna basin, which had the lowest average yield compared to all other FPUs. Adopting high yielding cultivars is projected to result in increased yields in all the basins except the Ganges and Indus basins but to a lesser extent, ranging between 5% in the Tapti basin and 11% in the Godavari basin compared to baseline. The application of critical irrigation during the pod filling stage for groundnut grown under rainfed conditions is projected to result in increased yields in all FPUs except for the Ganges and Indus basins compared in 2050. The increase is projected to be lesser than both the other adaptation options and ranges between 3% in the Tapti basin and 15% in the Krishna basin. None of the adaptation options are estimated to improve yields than baseline in 2050 in the Ganges and Indus basins (Fig. 5). The low yield response to the critical irrigation option in these two FPUs is mainly because water is not a limiting factor in the northern part of India.

Groundnut production in India was higher under all adaptation options in all the FPUs with the exception of the Ganges and Indus basins in 2050 under the GFDL-ESM2M scenario. The increase in production ranged between 18% in the Tapti basin and 39% in the Krishna basin compared to baseline in 2050. Adopting to higher yielding cultivars is projected to increase production in all the basins, but at a lower extent ranging between 8% in the Tapti basin and 19% in the Godavari basin compared to baseline. Providing one irrigation is also projected to result in increased yields in all the FPUs except for the Ganges basin compared to baseline. The scale of increase is projected to be lesser than both the other adaptation options and ranges between 6% in the Tapti basin and 24% in the Krishna basin (Table 3).

#### 3.4.4 Groundnut prices

**Supplementary Fig. 3:**
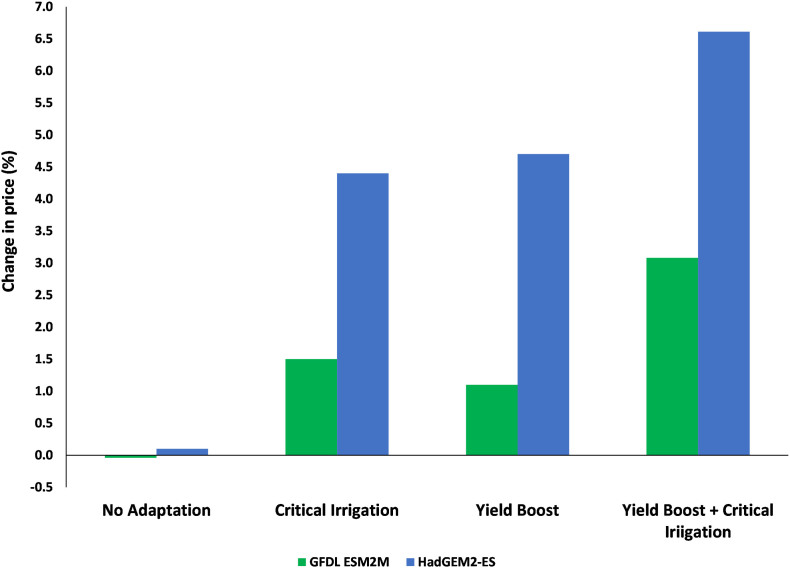
Percentage deviation in the producer price of groundnut in India in 2050 under various adaptation strategies.

**Supplementary Fig. 4:**
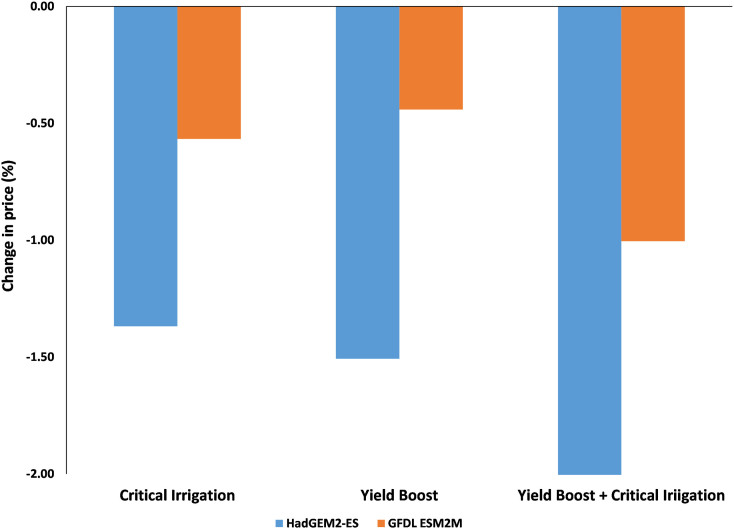
Percentage deviation in world price of groundnut from baseline under various adaptation strategies in 2050.

Changes in groundnut production in India lead to price fluctuations. All the adaptation scenarios allow producers to capture a greater share of the final consumer price. This leads to a situation where producer prices in India increase by between one to 3% under the GFDL-ESM2M scenario and by between four to 7% compared to baseline prices by 2050. This is seen as a natural consequence of adaptation and a decline in the global price of groundnut between 0.4% and 0.8% under the GFDL-ESM2M scenario and between 1.2% and 2% under the HadGEM 2-ES scenario compared to the baseline by 2050 (Supplementary Fig. 3, Supplementary Fig. 4).

## 4 Discussion

In this study we assessed (1) the potential impact of climate change on groundnut production in India during 2050 and an adaptation scenario where we tried different irrigation and varietal management options (2) we also combined climate and crop models with economic models to examine the impact of climate change not only on agricultural production but also on area, production, consumption and prices of groundnut. The uniqueness of this study lies in its efforts to link process-based crop simulation models with economic models to understand climate change complexities. By linking various socioeconomic scenarios that represent key drivers of future change, such as gross domestic product (GDP), population, and market effects, we undertook an *ex-ante* assessment of adaptation options on groundnut production and price.

In the current study, we chose two extreme climate change scenarios - one driest and another wettest to test the performance of various cultivars and management related adaptation options. This allowed us to observe the impacts of both pessimistic and optimistic climate scenarios. To reduce the negative impacts of climate change and to take the advantage of positive impacts, we identified several adaptation options based on in-depth literature reviews and consultations with scientists and experts in traditional management practices, modern farm applications, and new crop cultivars. We used crop simulation models to forecast crop growth and yield advantages due to new technologies in different FPUs. The models provide an opportunity to understand the impact of modifying various cultivar traits within the observed limits of their genetic variability and to assess the potential benefit of incorporating such traits (Boote et al., 2001; Singh et al., 2012).

We observed that projected reductions in groundnut yields due to climate change by 2050 are larger for some FPUs under pessimistic climate scenario and positive for the majority of FPUs except the Ganges basin under optimistic scenarios. The positive response is mainly due to increased mean annual rainfall amounts and CO_2_ fertilization. Further high year to year variation in rainfall coupled with high intensity rainfall events in the Ganges basin might have resulted in reduction in groundnut yields. The pessimistic scenario also shows some yield increase in the Godavari, Luni, and Krishna basins and negative impact in the Ganges, Indus, and Tapti basins. The negative impacts in these FPUs were attributed mainly due to relative high increase in both minimum and maximum temperatures compared to baseline scenarios in other FPUs (Supplementary Fig. 4). Williams and Boote, 1995; Weiss, 2000 found that optimum temperature for quick vegetative development in groundnut was between 25 and 30 °C and 35 °C for flower appearance and pegging (Prasad et al., 2003). Both GCMs predict an increase in mean maximum and minimum temperatures of more than 3–4 °C during the crop growth period. Several temperature tolerance studies in various field crops showed that increased temperatures during crop growth accelerates phenological development and advances the flowering date (Tao et al., 2009, Wang et al., 2015, Ye et al., 2020). Similarly, in groundnut, high temperatures might have resulted in faster crop senescence and poor seed filling due to increased demand for sink for assimilates because of other yield-limiting conditions (Kumar et al., 2012;Singh et al., 2014a, Singh et al., 2014b). In the current study, analysis of model output data across locations revealed that change in the duration of crop growth phases and hastening of crop maturity, decrease in the number of pods per plant and seed size were the main reasons for low yields in groundnut.

Further in majority of locations in India, groundnut is grown under rainfed conditions during monsoon season. Whenever a dry spell occurs, crop is exposed to drought conditions during the growth period. Drought conditions affect leaf expansion, photosynthesis and pod filling period which in turn affect shelling percentage (Clifford et al., 1993), Hence providing one critical irrigation and growing drought-tolerant cultivars will be very effective under these conditions. In addition, earlier studies also showed that with better root structure such as increased root length density, drought tolerant cultivars extract more water from increasing soil depth (Singh et al., 2012; Kadiyala et al., 2015).

Linking the economic and biophysical models allowed us to the factor in market effects, which is critical for trade-off analysis between economic and environmental parameters, *ex-ante* assessments of technologies and adaptation strategies, particularly in the long term (Islam et al., 2016; Nedumaran and Singh, 2017). The biophysical models involve technology and climate, and are essentially independent of prices, representing “non market” effects. The economic model captures markets and prices, which we call “market” effects (Islam et al., 2016; Robinson et al., 2015a, Robinson et al., 2015b).

In the economic model the yield growth over time consist of two components like exogenous and endogenous yields. The exogenous yields estimated with the economic model are affected by climate change, crop water availability and assumption of yield growth due to technology development but all these are independent of market effects. The endogenous yields are estimated within the economic model by considering the interactions of prices and trades with agriculture productivity (Robinson et al., 2015a, Robinson et al., 2015b). The price fluctuations due to changing yields and production in the economic model will reduce both the positive and negative impacts on crop yields by influencing farmer's decision to adjust farm inputs and management (Table 2).

Market effects moderate the impacts of climate change through price mechanisms as evident from our study as there was reduction in negative impact of climate change (Table 2). IMPACT model includes a short-run (annual), endogenous, response of yields to changes in both input and output prices. When market effects are also taken into consideration, prices and trade interact with agricultural productivity worldwide, producing what are defined as endogenous yields (Robinson et al., 2015a, Robinson et al., 2015b). Agriculture–estimated elasticities are adjusted to represent a synthesis of average, aggregate elasticities for each region, given the income level and distribution of urban and rural population. Over time the elasticities are adjusted to accommodate the gradual shift in demand from staples to high-value commodities like meat, especially in developing countries. This assumption is based on expected economic growth, increased urbanization, and continued commercialization of the agricultural sector.

Ceteris paribus, increased production reduces price and decreased production increase the price. In this study, under optimistic scenario, the increase in groundnut yield and production decrease the prices due to market effects. Optimistic scenario positively impacts groundnut yields with market effects but lower than the non-market effect. Under pessimistic climate scenario, the decrease in groundnut yields and production in India leads to increase in groundnut price. So, the increase price provide incentive to the farmers to increase farm inputs which dampens the negative impacts on groundnut yields.

Under the current levels of production, both consumer and producer prices will increase in the future. Adaptation strategies will increase groundnut yields at the farm level and could reduce the unit cost of production. The higher price and reduced production cost will provide an incentive to farmers to adopt yield increasing technologies and management options on larger areas. This will result in increasing groundnut production in India. Further the economic model projected a growing consumer demand for groundnut (shelled), confectionery groundnut, and oils in India, other south Asian countries, and the rest of the world. India is the second-largest exporter of groundnuts and its products after China (Birthal et al., 2010), and Indian groundnuts are competitive in international markets (Reddy and Bantilan, 2012). It is reasonable to expect that Indian farmers will continue to expand groundnut production by adopting various adaptation options to increase production to partially offset/minimize the negative impacts of climate change on yields, and also capitalize on the growing demand in India and the rest of the world. This will help India retain its position in the international groundnut market. From this study, it was also evident that endogenous behavioral responses such as food demand, area and other production factors are found to be very critical in differentiating final yield impacts from initial exogenous climate shocks. Hence it is essential to combine climate, crop, and economic models to estimate changes in yields and other parameters. The final yields estimated under this study captured the soil, climate, and crop management interactions, as well as market effects. Including socio-economic parameters while studying the climate change impacts will help policymakers and international donors to better understand the consequences of targeted actions in their priority setting exercises. However, the study has some limitations as models are not very effective in capturing extreme weather events, changes in pests and diseases, and ozone levels (Lobell and Gourdji, 2012).

## 5 Conclusion

Our analysis combining biophysical and economic models facilitated the estimation of impacts of different groundnut adaptation options to offset climate change impacts. The integrated assessment could guide policymakers to better understand the consequences of targeted actions in their priority setting exercises. The results clearly indicate the spatial and temporal variations in groundnut yields and production under climate change in India's groundnut growing regions and underscore the need for carefully targeted adaptation responses.

This study provides the first robust and comprehensive analysis based evidence that climate change impacts in a country like India have an impact on global prices, production and yields. Finally, it also helps international and national donors and policymakers to make informed decision-making. It encourages the public and private sectors to invest in climate change adaptation strategies in order to reduce the negative impacts of changing climate on agricultural food systems, build resilience and enhance smallholder farmers' income levels in developing countries where agriculture is the main source of livelihood.

The following are the supplementary data related to this article.

**Supplementary Fig. 5:**
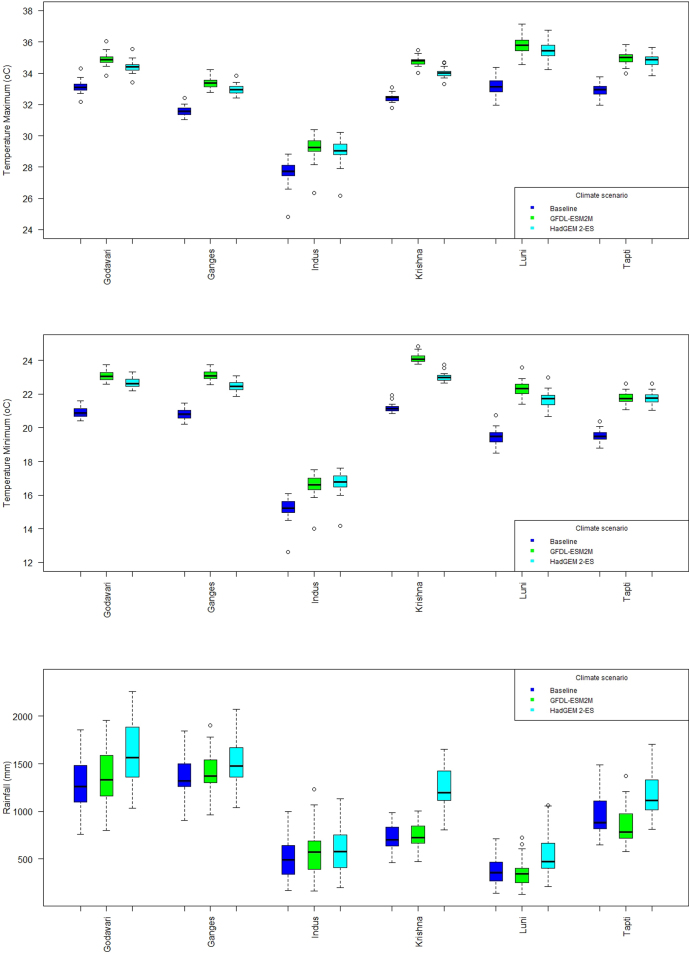
Projected mean (a) maximum temperature (b) minimum temperature and (c) rainfall for RCP8.5 mid-century compared to baseline. The box-and-whisker plots show the spread of data across the year.

Supplementary tables Supplementary Tables

## CRediT authorship contribution statement

**MDM Kadiyala**: Conceptualization, crop model simulation, original draft preparation, **Nedumaran S**: Economic analysis with IMPACT model, **Jyosthnaa**: Economic data analysis, visualization and data curation, **Murali Gumma**: Remote sensing & GIS analysis, crop type mapping, **Sridhar Gummadi**: crop model analysis using cluster computing facility, **Srigiri**: Adaptation options finalization, stakeholder engagement, **Ricky Roberson**: Software, **Anthony Whitbread**: Reviewing and editing and overall supervision

## Declaration of competing interest

The authors declare that they have no known competing financial interests or personal relationships that could have appeared to influence the work reported in this paper.
