# Supplementary material for: Modeling the potential impacts of climate change and adaptation strategies on groundnut production in India
Source: Sci Total Environ. 2021 Jul 1;776:145996. doi: 10.1016/j.scitotenv.2021.145996 (PMC8111332; doi:10.1016/j.scitotenv.2021.145996)
Supplement: Supplementary file 1 — Supplementary tables [file mmc6.docx]

Supplementary Table 1. Impact of different adaptation strategies on percent rainfed groundnut yield changes across various food producing units under climate change in India.

| FPU | Adaptation options for future climate (2040-2069) | | | | | |
| --- | --- | --- | --- | --- | --- | --- |
|  | HadGEM 2-ES | | | GFDL-ESM2M | | |
|  | Yield boost | Critical irrigation | Combination (CI+YB) | Yield boost (YB) | Critical irrigation (CI) | Combination (CI+YB) |
| Ganges | 19.9 | 0.8 | 21.0 | 20.7 | 10.2 | 33.3 |
| Godavari | 17.7 | 6.4 | 25.0 | 17.0 | 21.2 | 41.5 |
| Indus | 18.9 | 5.6 | 25.8 | 15.8 | 13.8 | 32.9 |
| Krishna | 16.4 | 24.9 | 44.9 | 14.9 | 34.8 | 54.2 |
| Luni | 17.4 | 1.9 | 19.6 | 16.3 | 8.9 | 27.0 |
| Tapti | 18.0 | 1.6 | 20.0 | 17.8 | 14.2 | 34.4 |
| Average | 18.1 | 6.9 | 26.1 | 17.1 | 17.2 | 37.2 |

Supplementary Table 2. GDP (billion 2005 US dollars) and population (million) assumptions of SSP 2 (Middle of the Road) scenario.

| Location | Gross Domestic product | | Population | |
| --- | --- | --- | --- | --- |
|  | **2010** | **2050** | **2010** | **2050** |
| World | 67,559 | 2,31,439 | 6,879 | 9,187 |
| South Asia | 4,461 | 32,939 | 1,630 | 2,373 |

Source: Robinson et al 2015

Supplementary Table 3: Pre-defined growth rates of socioeconomic drivers used in IMPACT for SSP2.

| Country | Gross Domestic product | | Population | |
| --- | --- | --- | --- | --- |
|  | **2010** | **2050** | **2010** | **2050** |
| India | 6.55 | 3.56 | 1.35 | 0.45 |

Source: IMPACT Model version 3.0

Supplementary Table 4: Key parameters and assumptions used in IMPACT model

| Parameter | Data Source | Explanation |
| --- | --- | --- |
| Demand elasticity | United States Department of Agriculture (USDA) and experts opinion | Determine demand responses to changes in prices and income. They have been adjusted over time to reflect changing preferences for high-value goods over staples due to economic growth. In addition, they are calibrated to be consistent with Engle’s Law, where food expenditure falls as a share of total expenditure with economic growth |
| Supply elasticity | Experts opinion | Determine production response to change in commodity response |
| Population growth rates | SSP database | IMPACT is calibrated to the International Institute for Applied Systems Analysis (IIASA) SSP 2 population Scenario. |
| GDP growth rates | SSP database | IMPACT is calibrated to the Organization for Economic Co-operation and Development (OECD) SSP 2 GDP scenario. |
